# From Precision Dosing to Acceptability: Customized Pharmaceutics and Innovations for Diverse Populations

**DOI:** 10.3390/pharmaceutics18101267

**Published:** 2026-10-07

**Authors:** Marina Delanni Vitória Guedes, Renata Vidor Contri, Irene Clemes Külkamp-Guerreiro

**Affiliations:** Graduate Program in Pharmaceutical Sciences, Federal University of Rio Grande do Sul, Porto Alegre 90610-000, RS, Brazil; marina.vitoria@ufrgs.br (M.D.V.G.); renata.contri@ufrgs.br (R.V.C.)

## 1. Introduction

Historically, pharmaceutical research and manufacturing have been predominantly driven by a “one-size-fits-all” paradigm, leading to a significant therapeutic gap for diverse patient demographics [1,2,3]. The unique physiological and metabolic requirements of patients, particularly in pediatric and geriatric populations, frequently render conventional adult dosage forms inappropriate or unsafe [1,2]. Consequently, healthcare professionals and caregivers are often forced to resort to extemporaneous compounding, such as crushing tablets. However, the manipulation of available solid dosage forms can lead to drug loss and potential tube blockage, thereby severely compromising patient safety and treatment efficacy [4]. In response to this critical scenario, recent developments have heavily underscored the absolute necessity of creating patient-centric dosage forms that effectively reconcile accurate dosing with palatability and ease of administration [1,2,5].

Building upon this clinical momentum, this Special Issue, “Customized Pharmaceutics: Innovations for Diverse Populations”, directly addresses these critical challenges by bringing together cutting-edge research on personalized medicine. Specifically, Contributions (1)–(5) collectively demonstrate how advances in additive manufacturing, alternative solid dosage forms, novel evaluation metrics, nanotechnology, and innovative lipid-based carriers exploiting specific patient diets are reshaping the landscape of drug delivery.

The shift toward point-of-care manufacturing, such as implementing 3D printing in hospital pharmacies, offers a promising path to magistral compounding and on-demand personalized medicine [3,6,7]. Taken together, these contributions reinforce the importance of integrating technological innovation with patient-centered design to overcome long-standing challenges in pharmaceutical development, ultimately advancing safer, more effective, and more accessible therapeutic solutions.

## 2. Overview of the Published Work

The transition from conventional to customized pharmaceutics requires a fundamental paradigm shift in both manufacturing technologies and formulation design. In this context, Contribution (1) provides a comprehensive review of how three-dimensional (3D) printing is transforming the pharmaceutical sector from traditional mass production to agile, on-demand additive manufacturing. By detailing various 3D printing techniques, such as fused deposition modeling (FDM), semisolid extrusion (SSE), and selective laser sintering (SLS), the authors demonstrate the technology’s ability to fabricate complex dosage forms, including multidrug polypills and formulations with customized release kinetics tailored to specific patient needs.

While 3D printing addresses the manufacturing of personalized doses, the physical format of the medication remains a critical factor in patient compliance. Mini-tablets have emerged as highly flexible and stable alternatives to liquid formulations, challenging conventional assumptions about solid dosage forms in pediatrics. This is addressed in Contribution (2), in which the authors conducted an extensive survey study to identify the maximum acceptable number of 2 mm and 3 mm mini-tablets among children, parents, and healthcare professionals. Their findings revealed that children and adolescents are willing to accept surprisingly high numbers of mini-tablets (ranging from 40 to 500 for the 2 mm size), even for middle- and long-term treatments, validating this approach as a highly acceptable strategy for precise dose titration.

However, the success of any oral pediatric formulation is intrinsically linked to its palatability. Contribution (3) addresses the lack of standardized methods for taste evaluation by introducing a novel metric adapted from the business sector, the Net Promoter Score (NPS) framework, which is repurposed here to calculate a Medicine Acceptability Score (MAS). Through a retrospective analysis of taste assessment data from multiple formulations, their study validated the MAS as a streamlined, single-metric tool capable of effectively discriminating between acceptable and unacceptable formulations, greatly simplifying the optimization phase in pharmaceutical development.

In the hospital setting, administration challenges extend beyond voluntary oral intake, particularly for patients who rely on enteral feeding. By developing an organic solvent-free formulation of phytantriol-based cubosomes to deliver nifedipine, Contribution (4) addressed the prevalent issue of tube obstruction and drug loss. When tested through pediatric nasogastric tubes of various calibers, these nanocarriers achieved superior flow rates and 100% drug recovery compared with extemporaneously crushed nifedipine tablets, offering a safer and highly efficient alternative for hospitalized pediatric patients.

Finally, the interaction between novel formulations and specific diets is a critical variable in diverse populations. The authors of Contribution (5) investigated the solubilization behavior of tolfenamic acid, a poorly water-soluble weakly acidic drug, when dispersed in reconstituted infant formula under simulated intestinal digestion. Unexpectedly, the results of this study demonstrated that the digestion process itself significantly increased the solubilization capacity for weakly acidic drugs, revealing an almost linear relationship between the amount of free fatty acids produced during lipolysis and the amount of drug dissolved. This highlights infant formula as a versatile lipid-based carrier capable of digestion-driven solubilization.

## 3. Future Perspectives

The research presented in this Special Issue highlights an irreversible shift in pharmaceutical sciences toward patient centricity. The demand for individualized therapies will continue to drive the integration of artificial intelligence in formulation design, optimizing processes such as 3D printing to autonomously predict drug release and stability. In this context, AI-driven algorithms and machine learning models hold tremendous potential for autonomously predicting drug release kinetics, optimizing excipient combinations, ensuring structural stability, and minimizing extensive trial-and-error testing. Furthermore, standardizing acceptability metrics and advancing nanotechnology for complex administration routes will be paramount. Ultimately, the future of medicine relies on our ability to translate these sophisticated, bench-top innovations into accessible, highly acceptable clinical realities for all patient populations.
